# Supplementary material for: The interactome of intact mitochondria by cross-linking mass spectrometry provides evidence for coexisting respiratory supercomplexes
Source: Mol Cell Proteomics. 2017 Dec 8;17(2):216–32. doi: 10.1074/mcp.RA117.000470 (PMC5795388; doi:10.1074/mcp.RA117.000470)
Supplement: Supplemental Data [file supp_RA117.000470_133922_0_supp_23978_fzffwf.zip › spectra_annotation/mito_DR_spectra_annotation/230-1-2-1-10-1.pdf]

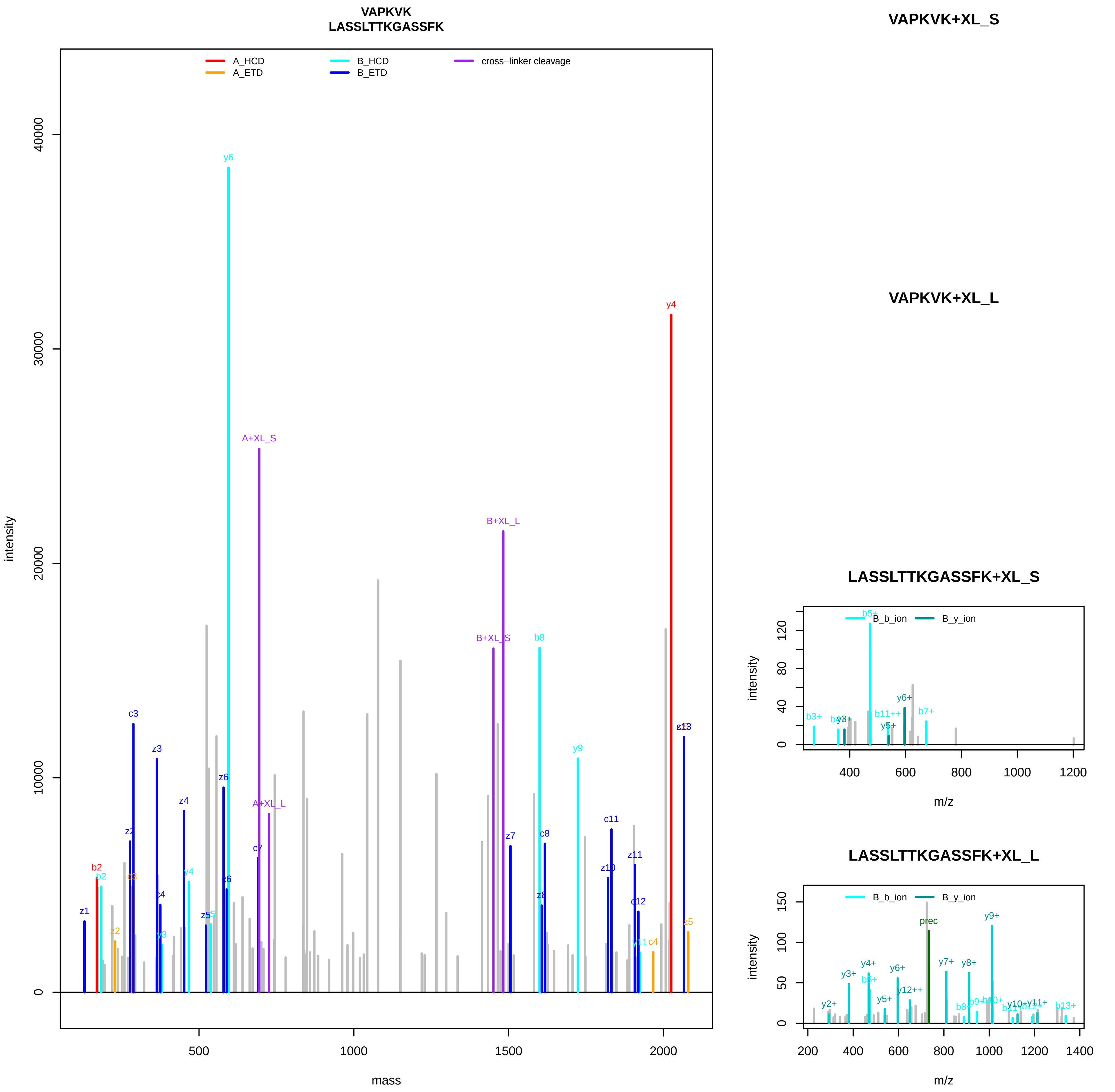

VAPKVK
LASSLTTKGASSFK
A_HCD
A_ETD
B_HCD
B_ETD
cross−linker cleavage
intensity
mass
y6
y4
A+XL_S
B+XL_L
B+XL_S
A+XL_L
b8
y9
c3
z3
z4
z6
c6
c7
z7
c8
c11
z10
z11
c12
z13
b2
z1
z5
c4
z5
0
10000
20000
30000
40000
500
1000
1500
2000
VAPKVK+XL_S
VAPKVK+XL_L
LASSLTTKGASSFK+XL_S
B_b_ion
B_y_ion
m/z
LASSLTTKGASSFK+XL_L
prec
y9+
y8+
y7+
y6+
y4+
y3+
y2+
b13+
